# Diagnosis of Primary Hepatic Lymphoma in a 55-Year-Old Male Patient Presented With Pain in the Right Hypochondrium: A Very Rare Case

**DOI:** 10.7759/cureus.25547

**Published:** 2022-05-31

**Authors:** Furqan Ul Haq, Said Amin, Hamza Yunus, Basharat Ullah, Wajid Ali

**Affiliations:** 1 Internal Medicine, Hayatabad Medical Complex Peshawar, Peshawar, PAK

**Keywords:** non-hodgkin's lymphoma, hepatic cancer, r-chop chemotherapy, duodenal diffuse large b-cell lymphoma, primary hepatic lymphoma

## Abstract

Primary hepatic lymphoma (PHL) is a rare subtype of diffuse large B-cell lymphoma (DLBCL). A 55-year-old male patient presented to us with jaundice and right upper quadrant pain. Investigations showed elevated alkaline phosphatase, lactate dehydrogenase, total bilirubin, normal alanine transaminase, and negative viral profile. Sonographic and computed tomographic scans show hepatosplenomegaly with hypodense lesion in liver associated with lymph nodes enlargement in the region of porta hepatis celiac axis, mediastinal and axillary lymphadenopathy. On immunohistochemistry, cells were positive for cluster of differentiation (CD)-19, CD-20, CD-21, c-myelocytomatosis oncogene (c-MYC), B-cell lymphoma 2 (Bcl-2), multiple myeloma oncogene-1 (MUM-1), same as B cell markers so it is diagnosed as PHL. DLBCL especially PHL shall be considered among the differentials of space-occupying lesions of liver. Early diagnosis of primary hepatic lymphoma is not a difficult task if excisional lymph node biopsy is taken following detection on ultrasound or CT scan which will lead to improved treatment, improvement in survival, and cost-effectiveness with good prognostic outcomes.

## Introduction

Primary hepatic lymphoma (PHL) is a hepatic presentation of diffuse large B-cell lymphoma (DLBCL). Among all non-Hodgkin's lymphoma (NHL) cases, the DLBCL comprises 30-40% of cases [1]. DLBCL is predominantly common in the sixth or seventh decade of life [2]. It presents itself in two forms - nodal DLBCL and extranodal DLBCL. The nodal DLBCL presents mainly in the lymph nodes (64.8%), Waldeyer’s ring (19.7%), mediastinum (12.8%), and the spleen (2.7%). Extranodal DLBCL involves stomach (22.4%), intestines (16%), nose and sinuses (8.9%), testis (8.4%), skin (7.9%), thyroid (6.9%), and CNS (6.4%), etc. while DLBCL involving the liver is a very rare type comprising only 0.4% [3]. Because of the very non-specific presentation of DLBCL involving the liver, its diagnosis becomes challenging clinically and the need for excisional biopsy of the liver and sub-hepatic lymph nodes is highly important in establishing a specific diagnosis. This case depicts this unique presentation involving the liver and emphasizes the role of clinical symptoms and histopathological features suggestive of hepatic DLBCL which will help in detecting the disease at earlier stages and improve the survival rate, cost-effective health care, and better long-term prognosis.

## Case presentation

This 55-year-old patient was presented on November 8, 2021, with swelling and non-colicky pain in the right hypochondrium. It was associated with intermittent low-grade fever, night sweats, and weight loss from the past four months. There was anorexia but no vomiting, loose stool, or pain aggravation on eating. The patient also reported generalized body weakness affecting his daily activities. His physical examination was remarkable for mild jaundice and pallor, mild-to-moderate tenderness in the right hypochondrium, hepatomegaly of 15.5 cm, and splenomegaly of 11 cm in its largest diameter. Investigations done are given below in Table 1.

**Table 1:** Laboratory outcomes for the patient. TLC: total leukocyte count; DLC: differential leukocyte count; RDW: red cell distribution width; HIV: human immunodeficiency virus; HCV: hepatitis C virus; HBsAg: hepatitis B surface antigen; ICT: information and communication technology; TSH: thyroid-stimulating hormone

S.n.	Entity of investigation			Results	Normal range
1.	TLC			5.63x10³/uL	4-11x10³/uL
2.	DLC	Neutrophils		77%	40-70%
		Lymphocytes		7.9%	20-25%
		Monocytes		14%	2-10%
3.	Hemoglobin			9.21 g/dL	12.5-16.5 g/dL
4.	Hematocrit			22.5%	36-54%
5.	RDW%			21.8%	11.5-14.5%
6.	Platelets			118x10³/uL	150-400x10³/uL
7.	Serum total bilirubin			2.4 mg/dL	0.1-1.2 mg/dL
8.	Alkaline phosphatase			312 u/L	44-147 IU/L
9.	Serum albumin			2.4 g/dL	3.4-5.4 g/dL
10.	Lactate dehydrogenase			341 U/L	140-280 U/L
11.	Serum uric acid			4.4 mg/dL	3.4-7.0 mg/dL
12.	Serum calcium			9.2 mg/dL	8-10 mg/dL
13.	Blood urea			42 mg/dL	18-45 mg/dL
14.	Creatinine			1.1 mg/dL	0.6-1.2 mg/dL
15.	Serum potassium			4.91 mmol/L	3.5-5.1 mmol/L
16.	Alanine transaminase			33 U/L	10-50 U/L
17.	Serum TSH levels			3.34 uIU/mL	0.3-4.2 uIU/mL
18.	Serum triglycerides			300 mg/dL	<200 mg/dL
19.	Cholesterol			228 mg/dL	<200 mg/dL
20.	Viral profile		Anti-HIV (ICT)	Negative	-
			Anti-HCV (ICT)	Negative	-
			HBsAg	Negative	-

**Figure 1:**
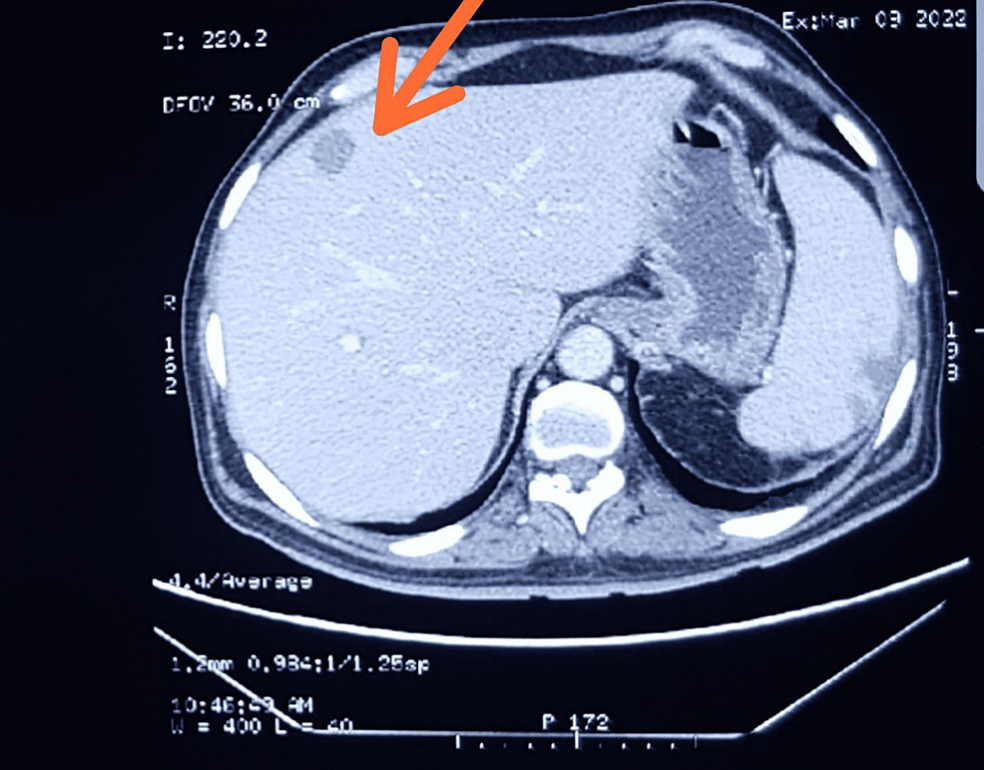
Hypodense lesion of 18x16 mm in segment 8 of liver as primary hepatic lymphoma (contrast in arterial phase).

**Figure 2:**
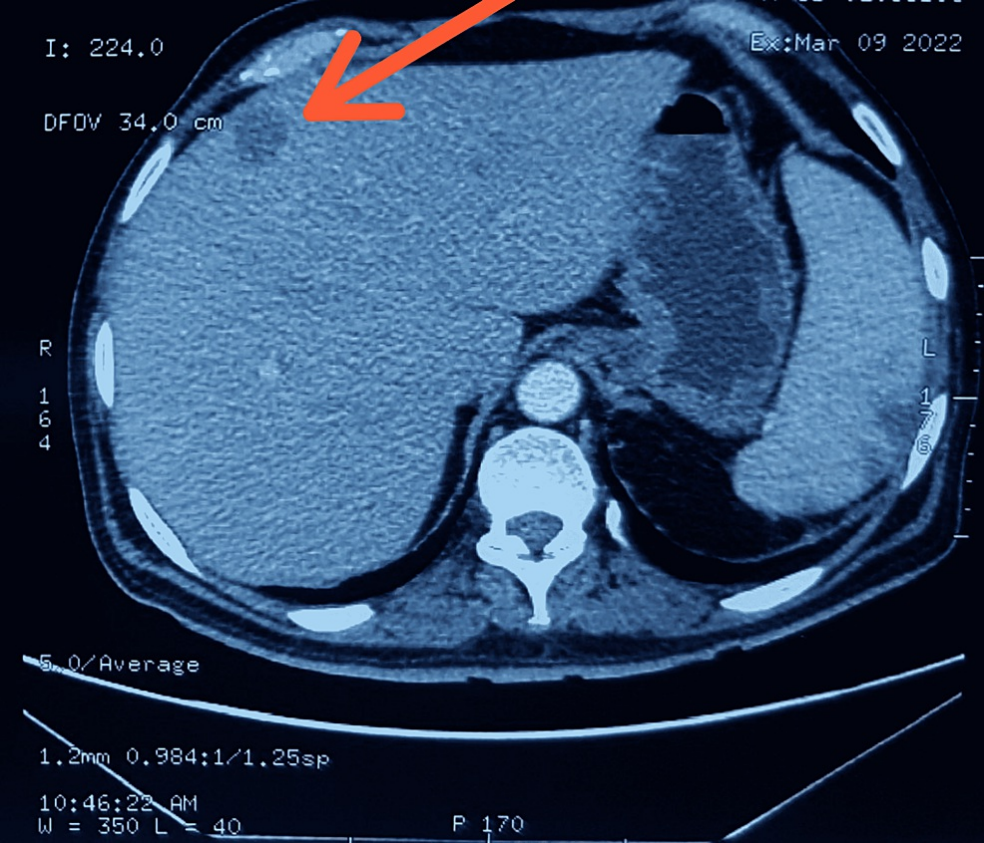
Hypodense lesion of 18x16 mm in segment 8 of liver as primary hepatic lymphoma (contrast in venous phase).

A CT scan of abdomen and pelvis (with contrast) was done on March 9, 2022, as described in (Table 2). This revealed enlarged liver and multiple enlarged lymph nodes; however, normal spleen, pancreas, and biliary tract. CT chest and neck done on March 9, 2022, showed multiple enlarged axillary and mediastinal lymph nodes (Figures 1, 2).

**Table 2:** CT abdomen and pelvis findings of the patient. IVC: inferior vena cava

S.n.	Structure	Impresion
1.	Liver	Enlarged measuring 16 cm in size, a hypodense, well-defined lesion measuring 18x16 mm was seen in segment 8 of the right lobe of liver.
2.	Spleen	Enlarged measuring 17.1 cm with wedge-shaped splenic infarcts.
3.	Lymph nodes	Multiple enlarged lymph nodes at the porta hepatis, celiac axis, peri-pancreatic, pre- and para-aortic, and pre-caval region were seen. Largest lymph node measuring 3.8x4.1 cm at porta hepatis is anteriorly compressing the second part of duodenum and posteriorly compressing and displacing IVC.
4.	Gallbladder and biliary tract	Normal

**Figure 3:**
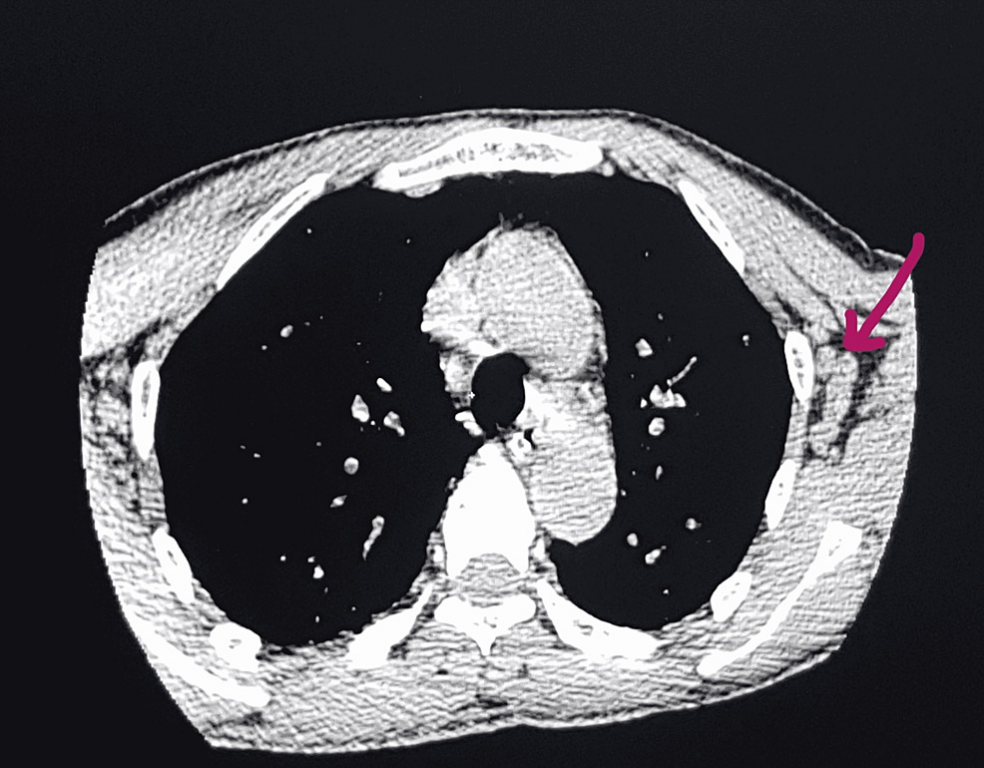
Multiple axillary lymph nodes in the left axillary region (arrow).

**Figure 4:**
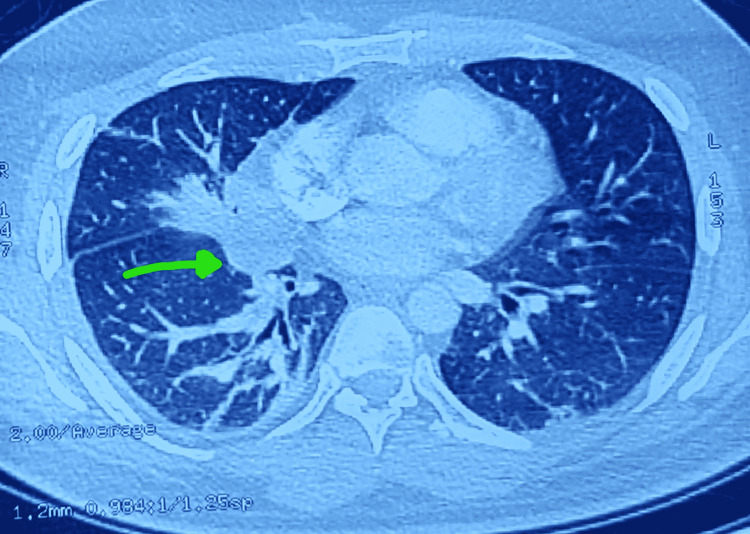
Mediastinal lymphadenopathy on chest CT scan (arrow).

CT scan chest and neck (metastatic workup) done on January 4, 2022, showed multiple enlarged axillary and mediastinal lymph nodes described in Table 3. Axillary lymphadenopathy and mediastinal lymphadenopathies are shown in Figures 3, 4.

**Table 3:** CT neck and chest findings of the patient.

S.n.	Structure	Impression
1.	Cervical lymph nodes	No cervical lymphadenopathy.
2.	Axillary lymph nodes	Enlarged bilateral axillary nodes were seen, the largest measuring 11.1 mm was seen in the right axilla.
3.	Mediastinal lymph nodes	Enlarged mediastinal nodes were seen, the largest measuring 19 mm was seen in the right para tracheal region.
4.	Lungs	Atelectatic bands were seen in both lungs; however, no nodules or mass noted.

**Figure 5:**
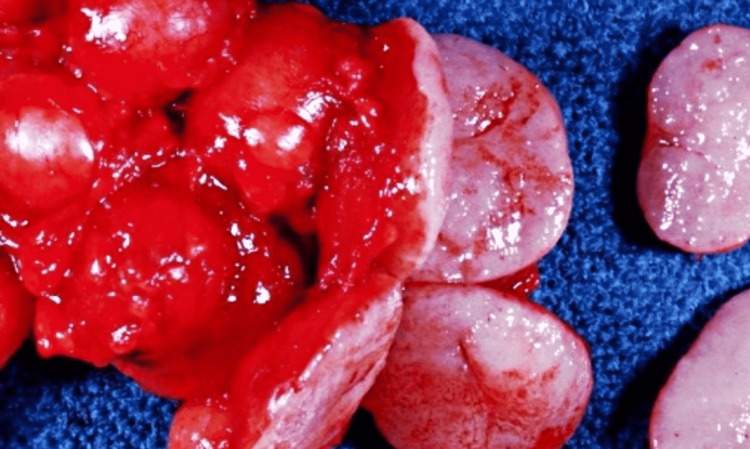
Gross sub-hepatic lymph nodes biopsy specimen received through excisional biopsy.

**Figure 6:**
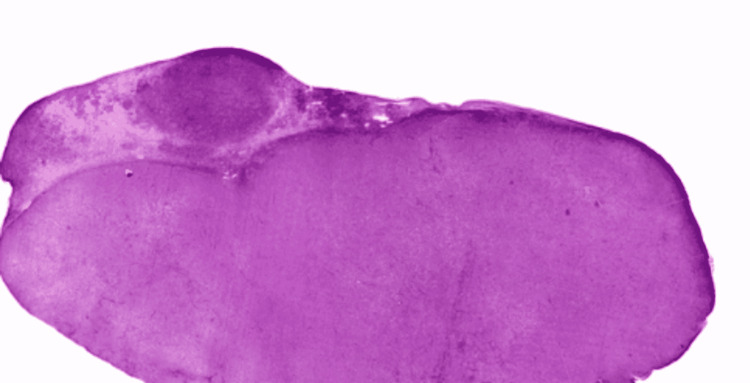
Low magnified LM specimen of lymph node of DLBCL. The image is showing diffuse infiltration of malignant lymphoid cells with disorganization of typical nodal architecture. In the upper left segment of the specimen, the tumor has invaded outside the capsule to soft tissues. DBCL: diffuse large B-cell lymphoma; LM: light microscopy

**Figure 7:**
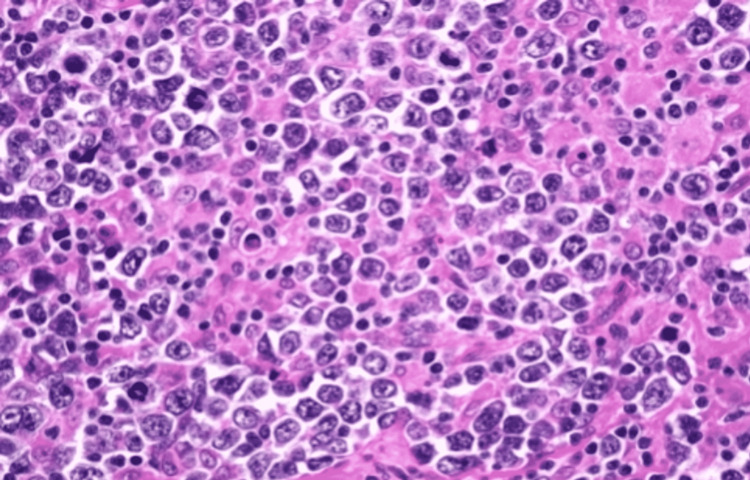
Magnified image of specimen showing diffuse large B-cell lymphoma. The image is showing nests of large to medium-sized neoplastic B cells having large nuclei (as compared to histiocytes and small lymphocytes), high nuclear-cytoplasmic (N/C) ratio, and having highly basophilic stained cytoplasm. These nests of cells are separated by a fibrillary matrix of stroma.

**Figure 8:**
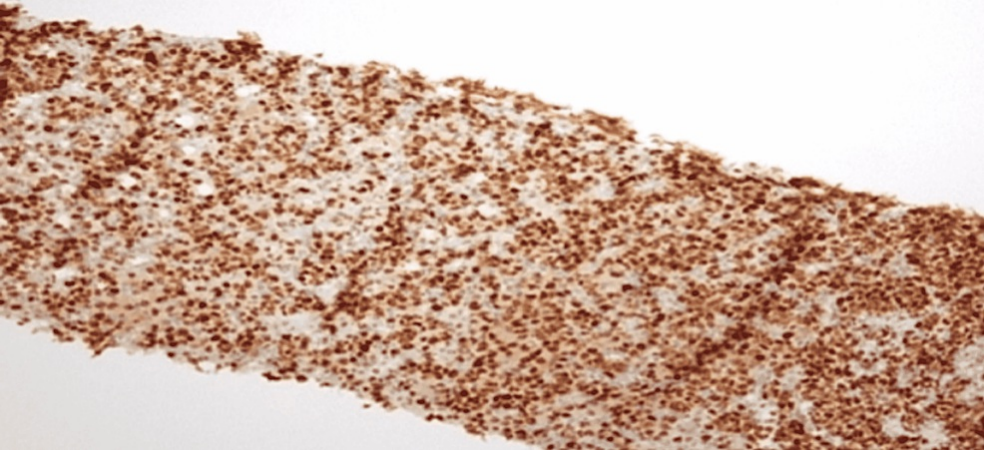
Bcl-6 stained diffuse large B-cell lymphoma (DLBCL) slide. Bcl: B-cell lymphoma

**Figure 9:**
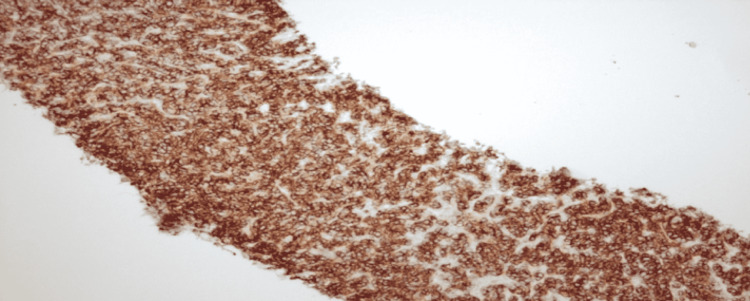
CD-20 stained diffuse large B-cell lymphoma (DLBCL) slide. CD: cluster of differentiation

Biopsy and histopathology reports are described in Table 4 and Figures 5-9 and have been labeled. Ultrasound-guided core biopsy of sub-hepatic lymph node was done and histopathology showed diffuse infiltrate of intermediate to large-sized round to oval cells with scanty cytoplasm and darkly stained nuclei with open chromatin and occasional prominent nuclei consistent with diffuse large B-cell lymphoma. Immunohistochemistry slides have been shown in Figures 1-7.

**Table 4:** Histopathology report findings of the patient. CD: cluster of differentiation; Bcl: B-cell lymphoma; MUM: multiple myeloma oncogene; c-MYC: c-myelocytomatosis oncogene

S.n.	Study	Impression
1.	Gross	Three tan-white colored cores and two core fragments measuring 0.5x0.2 cm.
2.	Microscopic	Diffuse infiltrates intermediate to large-sized round to oval cells with scant cytoplasm and darkly stained nuclei with open chromatin and occasional prominent nuclei consistent with diffuse large B-cell lymphoma.
3.	Immunohistochemistry	Immunohistochemistry showed CD-20, Bcl-2, Bcl-6, MUM-1, and c-MYC positive in 30% of cells and KI-7 in 70% of cells; however, CD-10 and CD-3 were negative.

Treatment and follow-up visit

The patient is on rituximab with dose-adjusted etoposide, prednisone, vincristine, cyclophosphamide, and doxorubicin (R-DA-EPOCH) therapy described in Table 5.

**Table 5:** Treatment received by the patient.

S.n.	Drug administered		Route	Dosage	Duration
1.	Rituximab (loading)		Intravenous	100 mg	1 day
2.	Rituximab		Intravenous	500 mg	1 day
3.	Etoposide		Intravenous	80 mg	4 days
4.	Deltacortil		Oral	5 mg	5 days
5.	Vincristine		Intravenous	0.6 mg	4 days
6.	Cyclophosphamide		Intravenous	1,200 mg	1 day
7.	Doxorubicin		Intravenous	16 mg	4 days
8.	Dexamethasone		Intravenous	4 mg/mL	1 day
9.	Omeprazole		Intravenous	40 mg	SOS
10.	Ondansetron		Intravenous	8 mg	SOS
11.	Gravinate		Intravenous	50 mg	SOS
12.	Home treatment	(1) Cefixime	Oral	400 mg	5 days
		(2) Amgofil	S/C injection	300 mg	3 days
		(3) Onset	Oral	8 mg	5 days
		(4) Omeprazol	Oral	40 mg	7 days
		(5) Tramal	Oral	50 mg	Continue
		(6) Nystatin drops	Oral	100,000 u/mL	5 days

The patient presented again on January 17, 2022, having completed his first cycle of chemotherapy on January 10, 2022, with fever and generalized weakness. His lab investigations at admission showed total leukocyte count (TLC) of 0.28x10^3^/uL with 10.9% neutrophils and 75% lymphocytes, hemoglobin of 6.8 g/dL, and platelets of 39x10^3^/uL. He was started on broad-spectrum antibiotics, supportive treatment, and three red cell concentrate (RCC) transfusions were done. On discharge from the hospital on March 20, 2022, his hemoglobin was improved to 8.16 g/dL, TLC to 3.39x10^6^/uL, with neutrophils 71% and lymphocytes 16%, while his platelets were raised to 54x10^3^/uL. Apart from slightly raised bilirubin of 2.8 mg/dL, his other laboratory reports including renal function tests were normal. 

Signs and symptoms of primary Hodgkin's lymphoma

Non-Hodgkin's lymphoma has symptoms such as fever, weight loss, abdominal pain, chest pain due to enlargement of lymph nodes in mediastinum, swollen abdomen, early satiety, and mild cough. Sometimes it is associated with recurrent infections and easy bruising and fatigue [4,5]. DLBCL presents similarly to other subtypes of non-Hodgkin's lymphomas but with some additional symptoms. These additional symptoms are variable and depend upon the type of DLBCL, its stage, and number of cycles of chemotherapy.

## Discussion

Primary hepatic lymphoma (PHL) is a rare type of diffuse large B-cell lymphoma (DLBCL) comprising less than 1% of all extranodal DLBCL cases. Because of its rarity and unusual presentation, it is often clinically indistinguishable from other common diseases like malaria, hydatid cyst, viral hepatitis, hepatocellular carcinoma, hepatic adenoma, and hepatic metastasis [6]. Investigations like alpha-fetoprotein and carcinoembryonic antigen levels may help differentiate PHL from hepatocellular carcinoma but these tests lack sensitivity and specificity [7,8]. Radiological imaging like ultrasound and computed tomography (CT) scans can reveal hypointense or hyperintense hepatic lesions and whether the lesion is unifocal, multifocal, or hepatic parenchyma is diffusely involved with coarse echotexture as in cirrhosis. However, like lab investigations, radiological imaging modalities too have limitations with regards to definitive diagnosis of PHL, which can be easily diagnosed with ultrasound or CT guided core biopsy of liver revealing diffuse infiltrates intermediate to large sized round to oval cells with scant cytoplasm and darkly stained nuclei with open chromatin and occasional prominent nucleoli.

Immunohistochemistry of biopsied specimen can further aid in diagnosing PHL which will show cluster of differentiation (CD)-20 positivity, B-cell lymphoma 2 (Bcl-2), Bcl-6, multiple myeloma oncogene-1 (MUM-1), c-myelocytomatosis oncogene (c-MYC) positivity in 30-50% cases, sometimes CD-19 and CD-21 as well that is consistent with B-cell markers [9,10]. Treatment primarily consists of chemotherapy with R-CHOP regimen that includes rituximab, cyclophosphamide, hydroxydaunorubicin, Oncovins, such as vincristine, prednisone, and sometimes etoposide [11]. Frequent follow-up visits for treatment response and management of chemotherapy-related side effects are crucial for improved health care outcomes including patient satisfaction and prevention of complications.

## Conclusions

One of the differentials for the space-occupying lesions in the liver is primary hepatic lymphoma if other malignancies are ruled out. Early diagnosis of PHL is not a difficult task if lymph node excisional biopsy is taken following detection on ultrasound or CT scan which will lead to early treatment initiation, early disease remission with improved long-term survival, and cost-effective health care.
